# Cardiovascular and Renal Risk Stratification in Patients Referred to an Onconephrology Clinic and Undergoing Different Oncology Therapies: A Real-World Study

**DOI:** 10.3390/biomedicines14061342

**Published:** 2026-06-13

**Authors:** Silvia Lai, Adolfo Marco Perrotta, Giovanni Pintus, Paolo Menè, Paolo Izzo, Sara Izzo, Lida Tartaglione, Luciano Izzo, Silverio Rotondi, Francesca Tinti, Luca Salomone, Anna Paola Mitterhofer, Simone Scagnoli, Andrea Botticelli, Daniele Santini, Giuseppe Ciniero, Alessandra Punzo, Gianluigi Zaza

**Affiliations:** 1 Nephrology Unit, Department of Translational and Precision Medicine, Policlinico Umberto I University Hospital, Sapienza University of Rome, 00185 Rome, Italy; silvia.lai@uniroma1.it (S.L.);; 2 Nephrology and Dialysis Unit, Department of Systems Medicine, University of Rome “Tor Vergata”, 00133 Rome, Italyannapaola.mitter@uniroma2.it (A.P.M.); 3 Nephrology and Dialysis Unit, Department of Clinical and Molecular Medicine, St. Andrea University Hospital, Sapienza University of Rome, 00185 Rome, Italy; 4 “Pietro Valdoni” Department of Surgery, Policlinico Umberto I University Hospital, Sapienza University of Rome, 00185 Rome, Italy; 5 Plastic and Reconstructive Surgery Unit, Multidisciplinary Department of Medical-Surgical and Dental Specialties, Università Degli Studi Della Campania “L. Vanvitelli”, 81100 Naples, Italy; 6 Department of Radiological Sciences, Oncology and Pathology, Sapienza University of Rome, 00185 Rome, Italy

**Keywords:** onconephrology, cardiovascular risk, acute kidney injury, chronic kidney disease, major adverse kidney events

## Abstract

**Background**: Onconephrology is an emerging field addressing renal and cardiovascular complications in patients with cancer. Kidney disease and cardiovascular risk frequently coexist and may significantly affect oncological outcomes. **Methods**: We conducted a single-center, prospective, observational study including adult oncological patients. Patients were evaluated at baseline and after 1, 3, and 12 months. Renal outcomes (Acute Kidney Injury (AKI), Chronic Kidney Disease (CKD), AKI on CKD, Acute Kidney Disease (AKD)), cardiovascular risk assessment scores (using the SCORE/SCORE2 systems), and major adverse kidney events (MAKEs) were recorded. **Results**: Eighty-three patients were enrolled (mean age 69.8 ± 11.6 years, 60.2% male). At baseline, AKI was present in 30.4%, CKD in 27.8%, and AKI on CKD in 16.5% of patients. Overall, 96.7% of the cohort was classified as having a high or very high cardiovascular risk. During follow-up, 18.1% experienced new AKI, and MAKEs occurred in 30.4% of patients, driven primarily by mortality. Male sex emerged as the main predictor of death. **Conclusions**: Onconephrology patients suffer from a high burden of renal disease and cardiovascular risk. Integrated nephrological and cardiovascular assessment may represent a key component of personalized cancer care.

## 1. Introduction

Advances in oncologic therapies have significantly improved patient survival and highlighted the importance of considering therapeutic approaches to co-existing chronic disease, particularly kidney disease. Acute kidney injury (AKI) and chronic kidney disease (CKD) are common complications among oncologic patients and may result from the malignancy itself, cancer-related comorbidities, or nephrotoxic anticancer treatments [1,2]. Importantly, kidney dysfunction not only impacts eligibility for and discontinuation of oncologic therapies but is also independently associated with increased cardiovascular (CV) risk and mortality [3].

The bidirectional relationship between cancer and kidney disease has spurred research in onconephrology, increasing the number of studies investigating preventive actions, early detection tools, and the management of renal complications. AKI is a frequent event in this population and is associated with adverse outcomes, including progression to CKD, recurrent kidney injury, major adverse kidney events (MAKEs), and death [4,5]. Moreover, patients with cancer and kidney disease often suffer from a high burden of traditional and non-traditional CV risk factors, further complicating clinical management [6,7].

Despite growing recognition of the clinical relevance of onconephrology, real-world data on clinical characteristics, renal outcomes, and CV risk in oncologic patients remain insufficient [8].

### Study Aims

The primary aim of this study was to evaluate the incidence of AKI.

The secondary aims were to evaluate long-term clinical outcomes in the population enrolled, to identify potential prognostic and predictive factors associated with these outcomes, to examine overall survival outcomes and assess CV risk, and to distinguish effects and recurrence in patients treated with immunotherapy and in those treated with targeted therapies.

Secondary endpoints included AKI recurrence, survival outcomes, and MAKEs, while CV risk assessment and subgroup analysis outcomes are explicitly presented as exploratory.

## 2. Materials and Methods

### 2.1. Study Design and Population

We conducted an observational, longitudinal, single-center cohort study involving consecutive patients with oncological disease followed at the oncology and nephrology outpatient clinics of Policlinico Umberto I between September 2024 and June 2025. Clinical and laboratory parameters (renal function, serum electrolytes, inflammatory markers, and arterial blood gas analysis and urinalysis) were assessed at baseline, prior to initiating oncologic therapy (T0). Evaluations occurred at T0, the baseline visit, and at T1, one month later; T2, three months later; and T3, twelve months later. Variables were predefined as either analytical variables or descriptive clinical variables. Pediatric patients, individuals with hematological malignancies, and patients undergoing renal replacement therapy and/or kidney transplantation were excluded. AKI and CKD were defined according to the KDIGO criteria. AKI was diagnosed based on changes in serum creatinine or urine output (KDIGO 2012). CKD was defined as estimated glomerular filtration rate (eGFR) < 60 mL/min/1.73 m^2^ and/or markers of kidney damage persisting ≥ 3 months (KDIGO 2024) [9,10]. In participants with pre-existing CKD, AKI was defined in accordance with the KDIGO criteria, using changes in serum creatinine relative to the individual baseline value. AKI was diagnosed as an absolute increase in serum creatinine of ≥0.3 mg/dL within 48 h or a relative increase of ≥50% from baseline within 7 days. Baseline kidney function was defined as the most recent stable outpatient serum creatinine measurement obtained prior to study initiation. When available, urine output criteria (<0.5 mL/kg/h for ≥6 h) were also applied. AKI severity was staged according to KDIGO guidelines. Acute kidney disease (AKD) is defined as the presence of any of the following functional or structural criteria in the appropriate clinical context with a duration ≤ 3 months: AKI (by functional criteria), eGFR < 60 mL/min/1.73 m^2^, decrease in eGFR by ≥35 mL/min per 1.73 m^2^ from baseline, serum creatinine increase >50%, or presence of marker of kidney damage (albuminuria, hematuria, or leukocyturia are most common) [4]. MAKEs were defined as a composite endpoint including (i) all-cause mortality, (ii) initiation of kidney replacement therapy, and (iii) a sustained decline in kidney function, defined as a ≥30% reduction in eGFR from baseline.

In patients with underlying CKD who developed AKI during the study period, management followed a standardized, guideline-based approach. This included systematic evaluation for pre-renal, intrinsic, and post-renal causes of kidney injury; prompt correction of hemodynamic disturbances; optimization of volume status; and discontinuation or dose adjustment of potentially nephrotoxic medications based on the eGFR. Renal function and electrolyte levels were monitored serially throughout the AKI episode [4]. According to international consensus in AKI research, including interpretations of the KDIGO criteria, renal recovery is defined as the return of kidney function toward baseline—reflected by normalization of or significant improvement in serum creatinine and eGFR—and independence from renal replacement therapy when previously required [11]. The study protocol was approved by the Clinical Research Ethics Committee of Sapienza, University of Rome, Italy and Policlinico Umberto I hospital, with Rif. 7641 and Prot. 0716/2024.

This study represents a real-world, single-center cohort of consecutive patients referred to a dedicated onconephrology outpatient clinic during the study period. The sample size was therefore determined by clinical referral activity and available cases rather than a predefined sample size calculation. Given the observational and exploratory nature of this study, no formal power analysis was performed. Subgroup analyses should thus be interpreted as hypothesis-generating.

### 2.2. Cardiovascular Risk Assessment

CV risk was assessed using the SCORE system (for patients aged 35–65 years), SCORE2 (for patients with diabetes), and SCORE2–Older Persons (for patients aged >65 years) [12,13]. SCORE values were calculated using the European Society of Cardiology online application, entering the following variables: age, sex, systolic blood pressure, total cholesterol, LDL cholesterol, HDL cholesterol, and smoking status. In patients with diabetes, HbA1c and the eGFR were also considered. Patients were directly classified as high risk for CV if they had severe hypertension, diabetes without CV risk factors and without target organ damage, or moderate CKD (GFR 30–59 mL/min/1.73 m^2^). Patients were considered to be at a very high risk of CV if they had documented CV disease, previous myocardial infarction, previous acute coronary syndrome, previous ischemic stroke, peripheral arterial disease, diabetes with one or more CV risk factors and/or markers of organ damage, or severe CKD (GFR < 30 mL/min/1.73 m^2^).

### 2.3. Additional Variables

The following variables were also collected: primary malignancy, presence of metastases at T0, duration of oncologic disease, oncologic therapy, and status of antineoplastic therapy. The following factors were considered when determining the antineoplastic therapy status: at T0, maintenance, discontinuation, or dose reduction; from T1 to T3, subsequent discontinuation, dose reduction, or a change in treatment line; mortality after T0; disease progression from T1 to T3; and in patients receiving immunotherapy, the incidence of renal immune-related adverse events (irAEs), reported both as absolute numbers and as a proportion of total cases suspected by the referring oncologist.

### 2.4. Clinical and Laboratory Assessment

For clinical evaluation, major medical history data were collected, including smoking status, biometric data (age, sex, weight, and body mass index), blood pressure (mmHg), and heart rate (bpm).

The laboratory data described below were collected from blood and urine samples.

**Peripheral venous blood samples**: Serum creatinine (sCr)(mg/dL), blood urea nitrogen (BUN) (mg/dL), sodium (mmol/L), potassium (mmol/L), chloride (mmol/L), calcium (mg/dL), magnesium (mg/dL), phosphorus (mg/dL), uric acid (mg/dL), glucose (mg/dL), glycated hemoglobin (HbA1c, %), complete blood count with differential, iron profile with transferrin saturation (TSAT = serum iron/[transferrin × 1.42], %), C-reactive protein (mg/L), fibrinogen (g/L), total cholesterol (mg/dL), LDL cholesterol (mg/dL), HDL cholesterol (mg/dL), triglycerides (mg/dL), and total serum proteins (g/dL), measured by protein electrophoresis and serum albumin (g/dL).

**Spot urine samples**: Specific gravity, microscopic hematuria and leukocyturia (number per high-power field), and albumin-to-creatinine ratio (ACR).

**Twenty-four-hour urine collection**: Urine volume (L/24 h), urinary creatinine (mg/24 h), proteinuria (mg/24 h), sodium (mmol/24 h), potassium (mmol/24 h), phosphate (mg/24 h), and urea (g/24 h).

The eGFR was calculated using the CKD-EPI formula.

The measured GFR (mGFR) was estimated according to creatinine clearance (ClCr).

### 2.5. Statistical Analysis

Statistical analysis was performed using SPSS Statistics for Windows (version 28; IBM, Chicago, IL, USA). Normality of data distribution was assessed using the Shapiro–Wilk test. Continuous variables were expressed as means and standard deviations (SDs), whereas categorical variables were reported as absolute numbers and percentages of the total. The Mann–Whitney U test was used to evaluate differences between continuous variables, while the chi-square test or Fisher’s exact test was applied to assess differences between categorical variables, as appropriate. Pearson’s or Spearman’s correlation tests were used to evaluate bivariate correlations. The association between variables and MAKEs and mortality outcomes was analyzed using logistic regression analysis. Multivariate Cox proportional hazards regression analyses were performed to investigate the effects of predefined variables on the MAKEs and mortality over time outcomes. Survival analysis was conducted using the Kaplan–Meier estimator, and the resulting curves were compared using the log-rank test. Missing data were not included in the analysis. A *p* value < 0.05 was considered statistically significant.

## 3. Results

### 3.1. Clinical Characteristics of the Onconephrology Cohort

In total, 83 patients, 50 males (60.2%) and 33 females (39.8%), with a mean age of 69.81 ± 11.56 years, were enrolled in this study. Table 1 provides a comprehensive summary of the participants’ baseline characteristics. The mean values of the biochemistry test results at T0, the first visit, were a mean sCr of 1.8 mg/dL (range 0.58–5.56) and a mean eGFR of 41.17 mL/min (10–107). Other mean values included serum uric acid, 7 mg/dL (2.7–12.6); total cholesterol, 220.33 mg/dL (117–474); HDL, 66.9 mg/dL (34–174); LDL, 135 mg/dL (68–193); Na+, 133 mmol/L (127–157); serum calcium, 9.21 mg/dL (2.35–10.60); potassium, 4.63 mmol/L (2.70–6); serum magnesium, 2.19 mg/dL (0.69–12.3); and serum phosphorus, 3.53 mg/dL (2–4.80). The majority of the population had a positive smoking history, whether they were active (18.9%) or former (56.5%) smokers. Comorbidities were prevalent: 71.3% of patients had arterial hypertension, 24.1% had type 2 diabetes mellitus, and 10.6% had a history of previous AKI episodes. The most common malignancies found in the study population were lung, kidney, and breast cancer, accounting for 28.6%, 15.7%, and 15.7% of the total, respectively. At the time of initial oncological diagnosis, 62.5% of patients presented with metastases; subsequently, in the interval between the initial diagnosis and the first nephrological evaluation, two additional patients experienced disease progression. At T0 (Table 2), AKI was present in 30.4% of cases—specifically, 67.6% of patients with AKI had stage 1, 18.9% had stage 2, and 13.5% had stage 3; CKD was present in 27.8% of cases (12% in stage 3a, 22.9% in stage 3b, 10% in stage 4, and 1.2% in stage 5); AKI on CKD was present in 16.5%; AKD was present in 10.1%; and other diagnoses (proteinuria in the non-nephrotic range and, in most cases, below 1 g/24 h, electrolyte imbalances, etc.) were present in 15.2% of cases (Table 3). In 30% of patients, discontinuation of therapy was necessary.

### 3.2. Cardiovascular Risk Assessment

Several CV risk factors were identified at T0, including advanced mean age, a high prevalence of smoking, diabetes mellitus, hypertension, and elevated mean levels of LDL, total cholesterol, and uric acid. CV risk was further stratified using the SCORE system: 3.3% of patients were at moderate risk, 63.9% were at high risk, and 32.8% were at very high risk.

### 3.3. Renal Function and Follow-Up Outcomes

**Figure 1:**
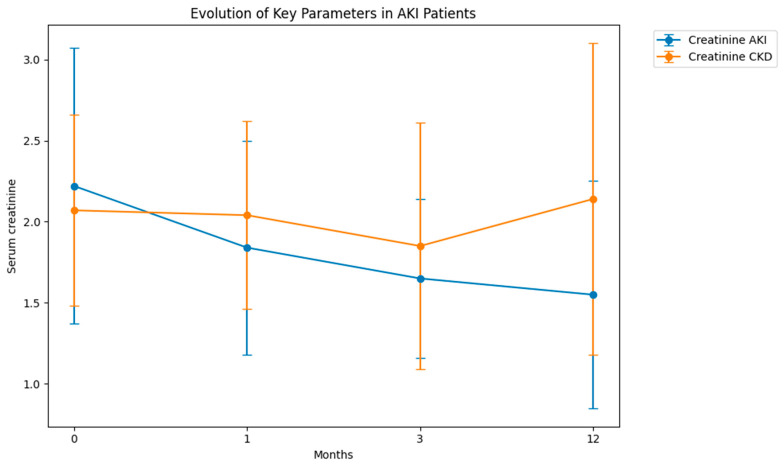
Evolution of serum creatinine in patients with AKI (blue line) and CKD (orange line). Abbreviations: AKI, Acute Kidney Injury; CKD, chronic Kidney Disease.

At the one-month evaluation (T1, median 29 days), sCr was significantly lower than baseline (1.74 mg/dL, *p* = 0.003), although three patients developed new-onset AKI (Figure 1). Mean uric acid at T1 was also significantly reduced compared with T0 at 6.37 mg/dL. At the three-month evaluation (T2, median 98 days), the mean sCr further decreased to 1.6 mg/dL (*p* = 0.002), despite six additional cases of AKI compared with T1. At the one-year follow-up (T3, median 357 days), the mean sCr was 1.46 mg/dL (*p* = 0.002).

Overall, over the entire follow-up period, 15 patients (18.1%) experienced a new AKI episode, while 28 patients (33.7%) showed oncological progression. At the end of the follow-up period, MAKEs occurred in 30.4% of patients; more than half of these (18.1% of the total cohort) were due to death, and all were related to oncological outcomes.

### 3.4. Analysis by Anticancer Therapy: ICPIs and Targeted Therapy

**Figure 2:**
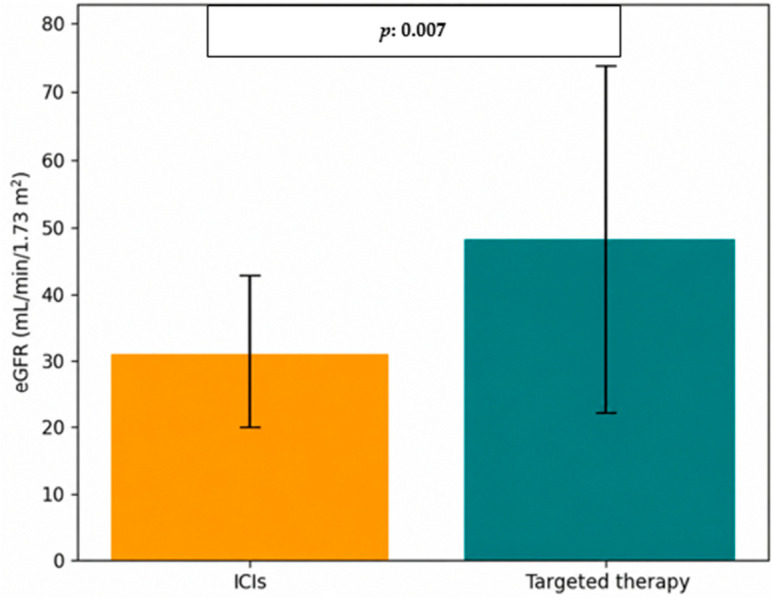
eGFR differences in patients treated with ICIs and targeted therapy. Abbreviations: ICIs, immune checkpoint inhibitors; eGFR, estimated glomerular filtration rate.

**Figure 3:**
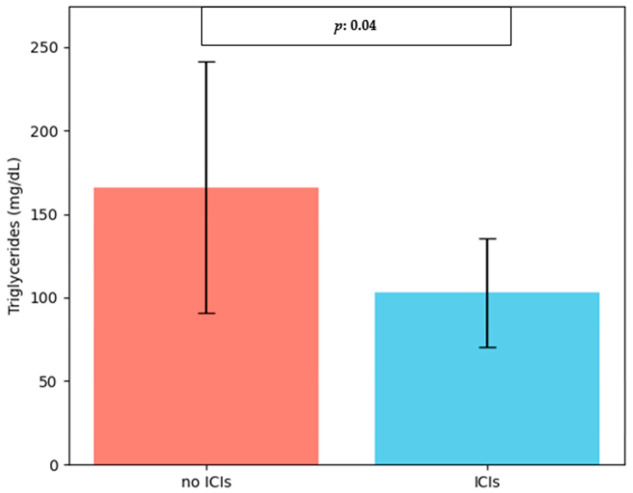
Triglyceride levels in patients treated with ICIs (blue) and no ICIs (red). Abbreviations: ICIs, immune checkpoint inhibitors.

The characteristics of patients receiving immune checkpoint inhibitors (ICPIs) versus targeted therapy (anti-VEGF) are detailed in Table 3, Table 4 and Table 5. In total, 27% of the cohort were treated with immunotherapy. These patients exhibited significantly higher mean creatinine and BUN levels compared with those treated with other therapies (sCr 2.27 mg/dL vs. 1.69 mg/dL, *p* = 0.003; urea 88.51 mg/dL vs. 53.81 mg/dL, *p* = 0.001) (Figure 2), while their triglyceride levels were significantly lower (102.85 mg/dL vs. 165.94 mg/dL, *p* = 0.046) (Figure 3). Patients on targeted therapy (anti-VEGF) (21% of the cohort) also showed significantly lower triglyceride levels (205 mg/dL vs. 127.52 mg/dL, *p* = 0.04). Four patients receiving both therapies were excluded from the comparative analysis.

Kaplan–Meier survival analysis for MAKEs and mortality (exitus) was performed for both drug classes. Various predictors—including CV risk (SCORE), immunotherapy, baseline diagnosis, recovery of renal function after AKI, new AKI episodes, hypertension, diabetes, and primary malignancy—did not show significant differences between survival curves. However, patients on targeted therapy showed improved survival, although not significantly, compared with those on immunotherapy (*p* = 0.080) and compared with the non-targeted therapy group (*p* = 0.055) (Table 6 and Table 7).

Logistic regression identified a significant association (*p* < 0.05) between male sex and mortality (14 males vs. 1 female) in both treatment groups. In the immunotherapy group, the initial AKI diagnosis was significantly correlated with the MAKEs outcome (*p* = 0.037). In the targeted therapy group, the presence of metastases at the first visit correlated with both MAKEs (*p* = 0.008) and mortality (*p* = 0.008). Cox regression analysis confirmed male sex as the only significant predictor of mortality (*p* = 0.029) and a marginal predictor of the MAKEs outcome (*p* = 0.072) (Table 8 and Table 9).

### 3.5. Analysis of the AKI Subgroup

The AKI subgroup (n = 38, Table 10) was predominantly male (57.9%), with diabetes in 24% and hypertension in 68% of cases. Regarding renal recovery, statistically significant reductions (*p* < 0.05) in sCr and BUN were observed from T0 to T3. sCr decreased from 2.22 mg/dL (T0) to 1.84 mg/dL (T1), to 1.65 mg/dL (T2), and to 1.57 mg/dL (T3), while BUN decreased from 75.28 mg/dL (T0) to 72.41 mg/dL (T1). During follow-up, 24% of patients developed new AKI, and 16% experienced disease progression. This subgroup presented a high CV risk at T0, characterized by advanced age, smoking, hypertension, diabetes, and elevated mean levels of uric acid (7.28 mg/dL), total cholesterol (203.25 mg/dL), and LDL (140 mg/dL).

### 3.6. Analysis of the CKD Subgroup

The CKD subgroup (n = 35, Table 11) was predominantly male (80%), hypertensive (85%), and diabetic (30%). There was a high prevalence of active (17%) and former (67%) smokers. At T0, 70% of patients had metastases, and 27% had already discontinued anticancer treatment. Eleven out of thirty-five patients developed at least one recurrent AKI episode. During follow-up, 34% (n = 12) experienced disease progression. According to the SCORE system, the presence of CKD automatically categorized these patients as high/very high risk. Additional CV risk factors included hypertension, diabetes, smoking, hyperuricemia (uric acid 7.87 mg/dL at T0), and hypercholesterolemia (total cholesterol 231.33 mg/dL at T2 and 207.50 mg/dL at T3; LDL 141 mg/dL at T0).

## 4. Discussion

In this study, we analyzed the clinical characteristics of an onconephrology population and, by describing their clinical phenotype, assessed their CV risk [14]. It is well-established that nephrological conditions such as CKD and AKI, as well as oncologic disease itself, represent independent CV risk factors [15]. Descriptive analysis showed that the outpatient population examined was predominantly elderly and male. This population exhibited a high prevalence of arterial hypertension and diabetes mellitus, which are among the main risk factors for the development of CKD. Furthermore, the high proportion of male patients, together with current or previous smoking habits, hyperuricemia, and elevated mean levels of total and LDL cholesterol, in association with the aforementioned comorbidities, resulted in a marked increase in CV risk. This finding was confirmed by SCORE assessment results, which showed that at baseline, 33% of patients were classified as very high risk and 64% as high risk. The predominance of male patients in our cohort may be partially explained by the increased incidence of cancer among males in Italy in recent years [16], as well as by the fact that male sex is a known risk factor for progression of renal damage [17,18,19]. Moreover, male sex, together with the presence of metastases at the first visit, emerged as a predictor of MAKEs and mortality outcomes in patients treated with either immunotherapy or targeted therapy. The observed association between male sex and mortality should be considered exploratory, given the limited number of events and the wide confidence interval, and therefore, it requires confirmation in larger studies.

One possible explanation is that the female population in our cohort was not only numerically smaller (less than one-third of the total) but was also largely composed of patients with breast cancer, who were receiving treatments with limited nephrotoxic potential.

At baseline, the prevalence of AKI was 30%, mainly stage 1 (67.6%), while the prevalence of CKD was 26.5%. During follow-up, a reduction in mean sCr values was observed among patients referred for AKI, likely reflecting the influence of multiple factors, including reduction or discontinuation of oncologic therapy and the implementation of nephroprotective strategies aimed at slowing renal damage progression (e.g., avoidance of NSAIDs, pharmacological and non-pharmacological management of hypertension and diabetes, low-protein and low-sodium diets). It is plausible that the significant reductions in sCr observed across all subgroup analyses (encompassing the overall population and patients with CKD, including AKI on CKD) were driven by recovery of renal function in patients with AKI. The aim of these interventions, shared with oncologists within a multidisciplinary management framework, were to not only achieve renal function recovery but also to slow CKD progression—considering the well-known bidirectional relationship between AKI and CKD—and reduce CV risk. The latter is supported by the statistically significant reduction in mean uric acid levels, a known marker of endothelial dysfunction and an important CV risk factor [20]. However, in an oncologic population, reductions in creatinine levels may not necessarily reflect true improvement in kidney function, as they can be influenced by factors such as muscle mass loss, cachexia, or cancer progression. Throughout the follow-up period, 15 patients developed a new episode of AKI, 28 experienced oncologic disease progression, and 15 died of cancer-related causes. Notably, despite interventions aimed at stabilizing renal function and the significant reduction in serum creatinine levels, nearly one-quarter of the cohort experienced recurrent AKI, which also emerged as an independent risk factor for mortality, as previously reported in the literature [21,22]. Although statistical power limitations prevented definitive conclusions, it is plausible that AKI—both as an initial diagnosis and as a recurrent or new-onset event—may contribute to disease progression in the long term, as it often necessitates reducing or discontinuing oncologic therapy [23]. Patients receiving immunotherapy exhibited more severely impaired renal function compared with those not receiving immunotherapy, as demonstrated by higher baseline sCr and BUN levels, as well as compared with those treated with targeted therapies [24,25]. In this subgroup, the presence of AKI was a major determinant of MAKEs and mortality outcomes, as reported by other authors [26]. Guven et al. reported an AKI incidence of 17.9% among 252 patients treated with ICIs, with baseline CKD, hypoalbuminemia, and RAAS inhibitor use emerging as independent risk factors, highlighting the need for better onconephrology collaboration and efforts to improve the nutritional status of ICI-treated patients [27]. In the absence of histological evidence, we speculate that these AKI episodes, characterized by a more unfavorable course, could be associated with acute interstitial nephritis with salt-wasting, as suggested by significantly lower mean sodium levels compared with other therapies [28].

In our cohort, patients receiving targeted therapy (anti-VEGF agents) showed higher levels of 24 h proteinuria compared with those not exposed to targeted treatments, although the difference did not reach statistical significance and remained within the non-nephrotic range. While this finding should be interpreted with caution, it is consistent with the known class effect of VEGF pathway inhibition on the glomerular filtration barrier. VEGF plays a critical role in maintaining endothelial cell integrity and podocyte–endothelial crosstalk; its inhibition may lead to endothelial dysfunction, glomerular injury, and increased permeability, clinically manifesting as proteinuria [12]. Interestingly, we also observed higher proteinuria in patients who were not receiving ICIs. Although this observation is exploratory, one possible explanation is that ICIs are less commonly associated with proteinuria compared with anti-VEGF agents and may instead induce different patterns of renal injury more frequently characterized by interstitial nephritis rather than glomerular damage. Alternatively, this finding may reflect differences in treatment allocation, underlying disease characteristics, or residual confounding [14]. Overall, these data are especially important for the interpretation of proteinuria as a marker of treatment-related renal phenotype, particularly considering the impact of proteinuria as a known CV risk factor. Patients treated with targeted therapy showed better survival compared with those receiving immunotherapy. This may be explained by the fact that immunotherapy, having been recently introduced into the oncologic setting, has been used as first-line therapy less frequently and is often administered in more advanced (i.e., metastatic) disease stages, thus involving patients with a higher baseline risk. In addition, patients receiving targeted therapy showed significantly higher mean triglyceride levels compared with untreated patients. Our first hypothesis is that there is possible class-related toxicity due to tyrosine kinase inhibitors (anti-VEGF), which are known to induce hypertriglyceridemia. However, we emphasize that the two groups are heterogeneous for cancer type, disease burden, and therapy line, and thus, we can only speculate about this class-related effect. This adverse effect warrants attention, as it may further increase CV risk, which is already elevated in this population. In this subgroup, the presence of metastases was correlated with MAKEs and mortality outcomes. The use of MAKEs and mortality as study endpoints allowed us to capture potential complications related to kidney disease occurring during the follow-up period. It should be emphasized that mortality represented a major component of the MAKEs outcomes and, in our cohort, was closely associated with oncologic disease progression, which may limit its specificity as a marker of kidney outcomes. Therefore, the high competing risk of death should be considered when interpreting these outcomes, and caution is warranted when inferring kidney-specific implications. This does not diminish the importance of nephrological clinical management or CV risk assessment; rather, it suggests that during the first year of follow-up, patients with advanced malignancy are at the greatest risk. Consequently, closer nephrological monitoring is required to ensure optimal antineoplastic therapy, with particular attention paid to avoiding prolonged treatment interruptions. Although the study population exhibited a high baseline CV risk, no CV-related deaths were observed during follow-up. However, CV data in this study were limited to baseline risk stratification, as CV events were not systematically recorded. Therefore, no conclusions can be drawn regarding CV outcomes. It is conceivable that with longer follow-up—which is now feasible due to improved survival associated with modern oncologic therapies—CV events may become more evident. Therefore, a multidisciplinary approach to the management of oncologic patients is essential, as it enables optimal control of comorbidities arising during cancer treatment [29]. Such an approach may help minimize therapy discontinuation or dose reductions secondary to renal complications, potentially improving long-term patient outcomes.

### Limitations

Our cohort consisted of a very select population of patients referred to our nephrology outpatient service, making the results difficult to generalize. The small sample size affected the statistical strength of our analysis and our conclusions. Larger studies are needed to thoroughly evaluate different drug-type-related effects in this complex population. Comparisons between treatment groups are limited by baseline clinical imbalances and the observational study design, raising the possibility of confounding by indication and precluding any causal inference regarding treatment effects. In our cohort, the MAKEs endpoint was largely affected by cancer-related mortality, which may limit its MAKEs ability to reflect kidney-specific outcomes and should be interpreted in the context of overall disease severity. CV events were not systematically recorded, limiting causal inference. The lack of systematic recording of CV events precludes any assessment of CV outcomes, limiting the analysis to baseline risk stratification. The limited number of events restricts the reliability of exploratory analyses of predictors of mortality, leading to imprecise estimates and potential overinterpretation.

Overall, these analyses should be regarded as exploratory and hypothesis-generating rather than confirmatory.

The 12-month follow-up period represents a limitation of this study, as it may not be sufficient to fully capture long-term renal and CV outcomes. Therefore, our findings primarily reflect short- to mid-term outcomes, and longer follow-up studies are needed to confirm and extend these observations.

In some patients, a paraneoplastic mechanism of renal involvement cannot be excluded; however, this could not be systematically evaluated due to the lack of histological and mechanistic data. Although all deaths in our cohort were clinically attributed to cancer progression, we cannot fully exclude the contribution of other causes; for this reason, the absence of a formal competing risk analysis may represent a limitation.

## 5. Conclusions

Patients referred to onconephrology clinics represent a high-risk population characterized by complex renal phenotypes and elevated CV risk. Early nephrological involvement and structured CV risk assessment may be essential components of personalized oncology care. This study’s population was characterized by increased CV risk, a high incidence of MAKEs and mortality, and a substantial risk of AKI recurrence. These results underscore the need for a multidisciplinary approach to the management of onconephrology patients and highlight the pivotal role of onconephrology in achieving the overarching goal of improving overall survival while preserving an acceptable quality of life.

## Figures and Tables

**Table 1:** Baseline characteristics of the onconephrology population. Data are expressed as means ± standard deviations or numbers (%).

Variables	Onconephrology Patients (n = 83)
Age (years)	69.8 ± 11.6
Sex, M/F, n (%)	50 (60.2)/33 (39.8)
BMI (kg/m^2^)	25.1 ± 5.3
SBP (mmHg)	128.6 ± 16.7
DBP (mmHg)	74.7 ± 9.8
Smoking status, %	
Current smoker	18.9
Non-smoker	24.6
Former smoker	56.5
Arterial hypertension, n (%)	59 (71.3)
Type 2 diabetes mellitus, n (%)	20 (24.0)
Serum creatinine (mg/dL)	1.8 ± 0.8
eGFR (mL/min/1.73 m^2^)	41.1 ± 21.2
BUN (mg/dL)	66.4 ± 33.8
Uric acid (mg/dL)	7.0 ± 2.2
Hemoglobin (g/dL)	11.6 ± 1.8
Total cholesterol (mg/dL)	220.3 ± 91.7
HDL cholesterol (mg/dL)	66.9 ± 39.3
LDL cholesterol (mg/dL)	135.8 ± 44.9
Sodium (mmol/L)	133.0 ± 4.5
Calcium (mg/dL)	9.2 ± 1.2
Potassium (mmol/L)	4.6 ± 0.6
Magnesium (mg/dL)	2.1 ± 1.9
Phosphorus (mg/dL)	3.5 ± 0.7

Abbreviations: SBP, systolic blood pressure; DBP, diastolic blood pressure; BUN, blood urea nitrogen; eGFR, estimated glomerular filtration rate; BMI, body mass index.

**Table 2:** Prevalence and stage of AKI and CKD at baseline (T0).

**AKI Stage**	**Prevalence (%)**
AKI 1	67.6
AKI 2	18.9
AKI 3	13.5
**CKD Stage**	**Prevalence (%)**
Stage 3a	12.0
Stage 3b	22.9
Stage 4	10.0
Stage 5	1.1

**Table 3:** Comparison of clinical characteristics between patients receiving and not receiving targeted therapy.

Variable	No Targeted Therapy (n = 57)	Targeted Therapy (n = 21)	*p* Value
24 h proteinuria (mg/24 h)	573.0 ± 544.4	547.2 ± 787.4	0.91
Age (years)	71.9 ± 11.0	65.5 ± 11.1	**0.02**
Serum creatinine (mg/dL)	1.9 ± 0.8	1.7 ± 0.7	0.32
eGFR (mL/min/1.73 m^2^)	38.3 ± 19.0	47.3 ± 25.8	0.09
Annual eGFR slope (mL/min/1.73 m^2^/year)	10.8 ± 24.3	6.8 ± 15.4	0.66
Serum urea (mg/dL)	67.8 ± 33.7	65.5 ± 37.6	0.41
Uric acid (mg/dL)	7.2 ± 2.1	6.4 ± 2.5	0.87
Total cholesterol (mg/dL)	187.0 ± 52.1	226.0 ± 139.4	0.28
LDL cholesterol (mg/dL)	104.2 ± 46.1	107.1 ± 47.4	0.09
HDL cholesterol (mg/dL)	58.8 ± 33.3	54.0 ± 13.4	0.70
Triglycerides (mg/dL)	127.5 ± 41.0	205.0 ± 118.8	**0.02**
Serum potassium (mmol/L)	4.6 ± 0.6	4.6 ± 0.7	0.76
Serum sodium baseline (mmol/L)	138.8 ± 4.4	141.2 ± 4.4	0.06
Serum sodium 3 months (mmol/L)	141.0 ± 2.0	141.6 ± 3.1	0.65

Abbreviations: eGFR, estimated glomerular filtration rate (CKD-EPI equation).

**Table 4:** Comparison of clinical characteristics between patients treated with immune checkpoint inhibitors (ICIs) and those not receiving ICIs.

Variable	No ICIs (n = 54)	ICIs (n = 27)	*p* Value
24 h proteinuria (mg/24 h)	695.2 ± 842.0	502.2 ± 476.0	0.43
Age (years)	68.6 ± 12.2	72.4 ± 9.9	0.17
C-reactive protein (CRP, mg/dL)	1.6 ± 12.2	2.3 ± 0.8	**0.003**
eGFR (mL/min/1.73 m^2^)	46.0 ± 22.7	31.9 ± 14.5	**0.004**
Annual eGFR slope	6.6 ± 2.0	14.1 ± 22.2	0.37
Serum urea (mg/dL)	53.8 ± 24.9	88.5 ± 34.5	**0.001**
Uric acid (mg/dL)	6.6 ± 2.5	7.6 ± 1.6	0.20
Triglycerides (mg/dL)	165.9 ± 75.4	102.8 ± 32.8	**0.04**

Abbreviations: ICI, immune checkpoint inhibitor; eGFR, estimated glomerular filtration rate; CRP, C-reactive protein.

**Table 5:** Comparison between patients receiving ICIs and targeted therapy.

Variable	ICIs (n = 27)	Targeted Therapy (n = 21)	*p* Value
Age (years)	73.3 ± 10.2	65.5 ± 11.5	**0.01**
Serum creatinine (mg/dL)	2.2 ± 0.9	1.7 ± 0.7	**0.02**
eGFR (mL/min/1.73 m^2^)	30.6 ± 10.6	47.8 ± 25.8	**0.007**
Sodium baseline (mmol/L)	137.9 ± 4.7	141.6 ± 3.1	**0.04**

Abbreviations: ICIs, immune checkpoint inhibitors; eGFR, estimated glomerular filtration rate.

**Table 6:** Kaplan–Meier analysis for MAKEs outcome: targeted therapy vs. no targeted therapy.

**Group**	**Total (n)**	**Events (n)**	**Censored (n)**	**Censored (%)**
No	34	17	17	50.0
Yes	13	2	11	84.6
Overall	47	19	28	59.6
**Mean Survival Time (Days)**			**SE**	**95% CI**
No: 343.6			22.4	299.8–387.5
Yes: 501.3			44.2	414.6–588.0

Log-rank (Mantel–Cox) χ^2^ = 3.67, df = 1, *p* = 0.055.

**Table 7:** Kaplan–Meier analysis for MAKEs outcome: immunotherapy vs. targeted therapy.

**Group**	**Total (n)**	**Events (n)**	**Censored (n)**	**Censored (%)**
Immunotherapy	13	6	7	53.8
Targeted therapy	13	2	11	84.6
**Mean Survival Time (Days)**			**SE**	**95% CI**
Immunotherapy			341.3	45.7
Targeted therapy			501.3	44.2

Log-rank test χ^2^ = 3.04, df = 1, *p* = 0.081.

**Table 8:** Cox regression for mortality outcome (Sex F = 0, M = 1).

Variable	B	SE	Wald	df	*p*	HR (ExpB)	95% CI
Sex (F = 0, M = 1)	2.288	1.051	4.740	1	0.029	9.852	1.256–77.249

Omnibus test: χ^2^ = 7.153, *p* = 0.007.

**Table 9:** Cox regression for MAKEs outcome (Sex F = 0, M = 1).

Variable	B	SE	Wald	df	*p*	HR (ExpB)	95% CI
Sex (F = 0, M = 1)	0.905	0.503	3.229	1	0.072	2.471	0.921–6.626

Omnibus test: χ^2^ = 3.436, *p* = 0.064.

**Table 10:** Evolution of key parameters in patients with AKI during follow-up.

Variable	T0	T1	T2	T3
Creatinine (mg/dL)	2.22 ± 0.85	1.84 ± 0.66	1.65 ± 0.49	1.55 ± 0.70
Urea (mg/dL)	75.28 ± 36.14	72.41 ± 30.65	61.40 ± 25.07	60.42 ± 30.63
Uric acid (mg/dL)	7.28 ± 1.81	6.14 ± 2.03	5.58 ± 1.41	6.24 ± 1.74
Sodium (mmol/L)	139.0 ± 3.60	140.0 ± 3.50	141.3 ± 4.10	141.0 ± 2.20
Potassium (mmol/L)	4.78 ± 0.65	4.62 ± 0.14	4.40 ± 0.40	4.53 ± 0.11
Calcium (mg/dL)	9.27 ± 1.49	9.60 ± 0.48	9.51 ± 0.39	9.66 ± 0.54
24 h proteinuria (mg/24 h)	860 ± 980	402 ± 335.3	418 ± 624	300 ± 100

**Table 11:** Evolution of key parameters in patients with chronic kidney disease during follow-up.

Variable	T0	T1	T2	T3
Creatinine (mg/dL)	2.07 ± 0.59	2.04 ± 0.58	1.85 ± 0.76	2.14 ± 0.96
Urea (mg/dL)	78.64 ± 26.20	77.41 ± 32.12	70.39 ± 29.26	89.70 ± 29.26
Total cholesterol (mg/dL)	169 ± 54.11	147.56 ± 21.67	231 ± 56.75	207 ± 73.34
HDL cholesterol (mg/dL)	94.33 ± 69.40	43.67 ± 12.43	66.00 ± 23.51	46.25 ± 2.98
Uric acid (mg/dL)	7.87 ± 2.37	7.11 ± 1.90	5.80 ± 2.06	6.40 ± 0.86
Sodium (mmol/L)	139.71 ± 4.07	139.59 ± 3.06	140.00 ± 4.34	141.00 ± 1.84
Potassium (mmol/L)	4.70 ± 0.50	5.01 ± 0.64	4.68 ± 0.53	4.55 ± 0.42
Calcium (mg/dL)	9.37 ± 0.80	8.78 ± 2.21	9.04 ± 1.16	9.78 ± 0.20

## Data Availability

The data presented in this study are not public due to privacy policy restriction but they are available on request from the corresponding author.
